# Role of the ArcAB two-component system in the resistance of *Escherichia coli *to reactive oxygen stress

**DOI:** 10.1186/1471-2180-9-183

**Published:** 2009-08-28

**Authors:** Cindy Loui, Alexander C Chang, Sangwei Lu

**Affiliations:** 1 Program in Infectious Diseases and Immunity, School of Public Health, University of California, Berkeley, CA 94720, USA

## Abstract

**Background:**

The global regulatory system ArcAB controls the anaerobic growth of *E. coli*, however, its role in aerobic conditions is not well characterized. We have previously reported that ArcA was necessary for *Salmonella *to resist reactive oxygen species (ROS) in aerobic conditions.

**Results:**

To investigate the mechanism of ROS resistance mediated by ArcAB, we generated deletion mutants of ArcA and ArcB in *E. coli*. Our results demonstrated that both ArcA and ArcB were necessary for resistance to hydrogen peroxide (H_2_O_2_), a type of ROS, and their function in this resistance was independent from H_2_O_2 _scavenge. Mutagenesis analysis of ArcA indicated that ROS resistance was mediated through a distinct signaling pathway from that used in anaerobic conditions. An abundant protein flagellin was elevated at both the protein and mRNA levels in the *ΔarcA *mutant as compared to the wild type *E. coli*, and deletion of flagellin restored the resistance of the *ΔarcA *mutant to H_2_O_2_. The resistance of the *ΔarcA *mutant *E. coli *to H_2_O_2 _can also be restored by amino acid supplementation, suggesting that a deficiency in amino acid and/or protein synthesis in the mutant contributed to its susceptibility to H_2_O_2_, which is consistent with the notion that protein synthesis is necessary for ROS resistance.

**Conclusion:**

Our results suggest that in addition to its role as a global regulator for anaerobic growth of bacteria, ArcAB system is also important for bacterial resistance to ROS in aerobic conditions, possibly through its influence on bacterial metabolism, especially amino acid and/or protein assimilation and synthesis.

## Background

Aerobic bacteria use oxygen as a terminal electron acceptor in oxygen-containing environments for their metabolism. Although aerobic growth has its obvious advantages (e. g. high energy efficiency, abundance of oxygen in the atmosphere, etc), bacteria must deal with the undesired consequences from exposure to oxygen and oxidative environments. Oxygen and its derivatives, such as superoxide and hydrogen peroxide, are often highly reactive and pose a threat to many macromolecules, such as enzymes with iron-sulfur centers, nucleic acids, and lipids. Therefore, bacteria undergoing aerobic growth must be able to sense, respond to, and detoxify reactive oxygen species (ROS), and maintain their structural and functional integrities.

The principle mechanism through which bacteria respond to environmental signals is through two-component and other regulatory systems [1,2]. At least four global regulatory systems -OxyRS, SoxRS, Fnr and ArcAB – are identified to respond to oxygen and its derivatives [3,4]. OxyRS and SoxRS systems control the response of bacteria to hydrogen peroxide and superoxide, respectively [3-12]. Fnr (fumarate and nitrate reduction) controls the transition from aerobic growth to anaerobic growth [13-17]. Fnr is believed to directly sense oxygen [18-20] and regulate at least 100 operons [21-23]. In addition to Fnr, the two-component regulatory system ArcAB also regulates the transition of bacteria from aerobic to anaerobic growth and is active under microaerobic conditions. It controls at least 100 operons that are involved in the TCA cycle and energy metabolism [16,24-29]. The sensor kinase ArcB undergoes auto-phosphorylation at His292 under anaerobic conditions, and this activation is negatively regulated by the oxidized quinones under aerobic conditions [25]. Activated ArcB undergoes a phosphorelay of His292 to Asp576 to His717, and subsequently activates its cognate transcriptional regulator ArcA by phosphorylating ArcA at Asp54 to repress genes contributing to aerobic metabolism (e.g. citrate synthase and isocitrate lyase) and activates genes necessary for anaerobic metabolism (e.g. pyruvate formate lyase and hydrogenase) [23,25,30-34].

Although the function of the ArcAB system in the anaerobic growth of *E. coli *has been well characterized, its function is unlikely to be limited to those required for the anaerobic growth of bacteria. For example, the ArcAB system has been reported to be involved in chromosomal replication, stress responses and aging of bacteria [35-37]. We have previously reported that ArcA of *Salmonella enterica *is necessary for its resistance to reactive oxygen and nitrogen species (ROS and RNS) [38]. More recently, ArcA is implicated in the ROS stress response of *Haemophilus influenzae *[39]. In this report, we analyzed the role of ArcAB in reactive oxygen resistance of *E. coli *and investigated the mechanism of ROS resistance mediated by the ArcAB two-component system.

## Results

### ArcAB system is necessary for *E. coli *to resist hydrogen peroxide (H_2_O_2_)

**Figure 1:**
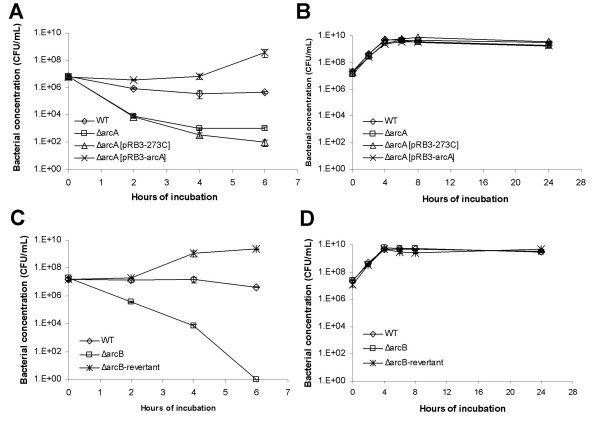
**Resistance of the *ΔarcA *and *ΔarcB *mutant of *E. coli *to H_2_O_2_**. (A and B) Growth and survival of wild type *E. coli *(diamond), *ΔarcA *mutant *E. coli *(square), *ΔarcA *mutant *E. coli *transformed with plasmid pRB3-273C (triangle) and *ΔarcA *mutant *E. coli *transformed with plasmid pRB3-arcA (cross) in LB broth with 1.5 mM H_2_O_2 _(A) or LB broth alone (B). (C and D) Growth and survival of wild type *E. coli *(diamond), *ΔarcB *mutant *E. coli *(square) and *ΔarcB *revertant mutant *E. coli *(cross) in LB broth with 1.5 mM H_2_O_2 _(C) or LB broth alone (D). Bacterial concentration was determined by plating and plotted against the indicated incubation time period. At least three experiments were performed, and results from a representative experiment performed in triplicates are shown. Error bars indicate standard deviation and sometimes fall within the data label.

To determine if the ArcAB global regulatory system plays a role in the survival of *E. coli *under stress by reactive oxygen species (ROS), we generated deletion mutants of ArcA (the global regulator) and ArcB (the cognate sensor-kinase of ArcA) in *E. coli *(Table 1). Both *ΔarcA *and *ΔarcB *mutant *E. coli *formed smaller colonies than their parental *E. coli*, but otherwise showed similar colony morphology. The *ΔarcA *and *ΔarcB *mutant *E. coli *were tested for their growth properties in complete (Luria Bertani broth) or minimal (M9) medium with glucose as carbon source. Overnight culture of each bacterial strain was diluted 1:100 in LB or M9 medium, and the growth of bacteria was measured by the optical density of the culture at 550 nm (OD_550 nm_) every 2 hours for 8 hours and then at 24 hours. This incubation period includes both log phase of growth and stationary phase of bacteria. We found that OD_550 nm _of both *ΔarcA *and *ΔarcB *mutants appeared to be lower than that of the wild type *E. coli *during the log phase of growth. However, both mutants had similar bacterial concentrations and growth curves to those of the wild type *E. coli *when their growth was quantified by plating (Figure 1B and 1D). Therefore, no gross defect was observed in *ΔarcA *and *ΔarcB *mutants in spite of lower OD_550 nm _of their cultures. The anaerobic growth of the *ΔarcA *and *ΔarcB *mutant *E. coli *was also tested and compared to that of the wild type *E. coli*. No defect was detected (data not shown). Similar results were obtained with LB broth and M9 minimal medium, results obtained with LB broth are shown (Figure 1).

**Table 1:** Bacterial strains, plasmids and oligonucleotides used for mutagenesis.

Bacterial strains and plasmids		Characteristics	Source or reference
*E. coli *strains	K12	Isolate MG1655	Dr. Sydney Kustu, University of California
	
	Δ*arcA*	Δ*arcA::kan *derivative of K12	This study
	
	Δ*arcB*	Δ*arcB::cm *derivative of K12	This study
	
	*arcB*::*kan*	derivative of K12 in which Kan^r ^was inserted adjacent to *arcB *while maintaining the function of *arcB*	This study
	
	Δ*arcB*-rev	*kan *derivative of Δ*arcB *with *arcB::cm *replaced by wild type *arcB*	This study
	
	Δ*fliC*	*fliC *non-polar deletion mutant of K12	This study
	
	Δ*arcA*/Δ*fliC*	Δ*arcA::kan/*Δ*fliC *derivative of K12	This study

Plasmids	pRB3-273C	Ap^r^, low to medium copy number plasmid	[40]
	
	pRB3-arcA	derivative of pRB3-273C containing *arcA*	[38]
	
	pRB3-arcD2A	derivative of pRB3-arcA containing Asp54 → Ala mutation	This study

**Oligonucleotides**	**Used for**	**Sequence**	

arcA5KO	mutagenesis of *arcA*	5'-tcttatcgttgaagacgagttggtaacacgcaacacgttg aaaagtattttcgaagcgga**gtgtaggctggagctgcttc**-3'	

arcA3KO	mutagenesis of *arcA*	5'-tcttccagatcaccgcagaagcgataaccttcaccgtgaa tggtggcgatgatttccggc**catatgaatatcctccttag**-3'	

arcB5KO	mutagenesis of *arcB*	5'-gccctcgtcgttcttgccattgtggtacaaatggcggtaaccatggtgct gcatggtcaggtcgaaagcattgatgttat**gtgtaggctggagctgcttc**-3'	

arcB3KO	mutagenesis of *arcB*	5'-gtggcttttgccacccacgctttcagcacttctacgtcgtgacgccactc ttctttcatctcttcaatccattcaccgac**catatgaatatcctccttag**-3'	

arcB-rev5	generation of *arcB*::*kan*	5'-cacattaatttttttaataaaaatggtacgcatcacacatttaactgattcatgtaacaa atcatttaagttttgctatcttaactgcgt**catatgaatatcctccttag**-3'	

arcB-rev3	generation of *arcB*::*kan*	5'-gcgaatactgcgccaacaccagggaaatcttggctgcgccgtaaattattatgatga gttacaagggcacagcactgtttttcaggccgc**gtgtaggctggagctgcttc**-3'	

fliC5KO	mutagenesis of *fliC*	5'-tcgctgatcactcaaaataatatcaacaagaaccagtctgcgctgtcgag ttctatcgagcgtctgtcttctggcttgcg**gtgtaggctggagctgcttc**-3'	

fliC3KO	mutagenesis of *fliC*	5'-ctgcggtacctggttagcttttgccaacacggagttaccggcctgctgga tgatctgcgctttcgacatattggacactt**catatgaatatcctccttag**-3'	

*kan*, kanamycin resistance cassette; *cm*, chloramphenicol resistance cassette. Sequences in bold in the table indicate those that are homologous to plasmids pKD3 and pKD4 [50], which were used as PCR templates for mutagenesis.

We assayed the resistance of the *ΔarcA *mutant *E. coli *to hydrogen peroxide (H_2_O_2_). Overnight culture of the *ΔarcA *mutant *E. coli *was exposed to H_2_O_2_, and its survival was compared to that of the wild type *E. coli*. The *ΔarcA *mutant *E. coli *was more susceptible than the wild type *E. coli *(Figure 1A). Plasmid pRB3-arcA, which carries a wild type allele of *arcA *in plasmid pRB3-273C [38,40], complemented the survival defects in H_2_O_2_. This indicates that the susceptible phenotype of the *ΔarcA *mutant *E. coli *was likely due to the deletion of the *arcA *allele (Figure 1A). Assays performed with log-phase culture of the *ΔarcA *mutant *E. coli *yielded similar results (data not shown). Similar results were obtained with LB broth and M9 minimal medium, results obtained with LB broth are shown (Figure 1).

The same analysis was carried out for ArcB, the cognate sensor-kinase of the ArcAB system. The *ΔarcB *mutant *E. coli *survived less than the wild type parental strain (Figure 1C). We had attempted to clone a wild type allele of *arcB *into plasmid pRB3-273C to complement the *ΔarcB *mutant *E. coli*. However, the cloning efficiency was unusually low as compared to similar cloning attempts we had conducted with the plasmid vector. Of a total of 7 recombinant plasmids we eventually obtained from several transformations, 5 contained mutations at the start codon of *arcB *and the remaining 2 had mutations that produced truncations early in the ORF (data not shown). This indicates that an over-expression of *arcB *from a plasmid is probably toxic to *E. coli*. As an alternative, we constructed a revertant of the *ΔarcB *mutant *E. coli*, in which a wild type *arcB *allele replaced the deleted *arcB *allele (see Materials and Methods). The revertant mutant of *ΔarcB *was shown to have the same resistance to H_2_O_2 _as the wild type *E. coli *(Figure 1C).

### The ArcAB system is dispensable for H_2_O_2 _scavenge

**Figure 2:**
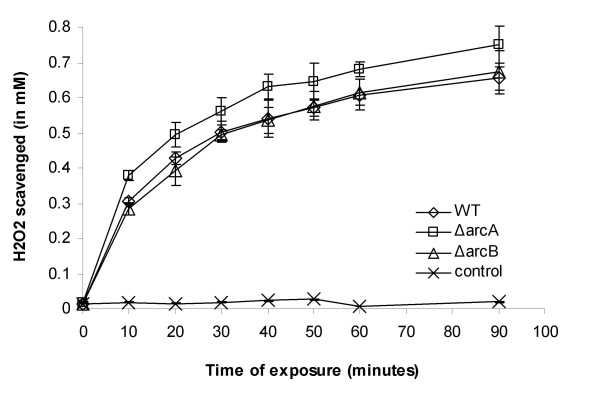
**The ArcAB system is dispensable for H_2_O_2 _scavenge**. The *ΔarcA *(square), *ΔarcB *(triangle) mutant and the wild type *E. coli *K12 (diamond) was cultured in LB broth supplemented with 2 mM of H_2_O_2 _at 37°C with shaking. Bacterial concentration and the H_2_O_2 _concentration were measured at various time points. The H_2_O_2 _scavenge was measured as the decrease of H_2_O_2 _concentration per 10^7 ^c.f.u. bacteria. A control sample without bacteria (cross) was included to monitor any possible spontaneous degradation of H_2_O_2_. The experiment was repeated at least three times, and data from one representative assay performed in duplicates were shown. Error bars indicate standard deviation and sometimes fall within the data label.

To determine the mechanism of how the ArcAB system is involved in H_2_O_2 _resistance, we analyzed the H_2_O_2 _scavenging activity of the *ΔarcA *and *ΔarcB *mutant of *E. coli *K12, since a defect in H_2_O_2 _scavenging activity may lead to the susceptibility to H_2_O_2_. The overnight culture was diluted in LB containing 2 mM of H_2_O_2_, and the concentration of the residual H_2_O_2 _was measured after various incubation period. The scavenge of H_2_O_2 _was measured as the reduction in H_2_O_2 _concentration over the incubation period. Our results indicate that both *ΔarcA *and *ΔarcB *mutants scavenged H_2_O_2 _normally as compared to the wild type *E. coli *K12., and no deficiency was observed (Figure 2).

### Phosphorylation at Asp54 is dispensable for H_2_O_2 _resistance mediated by ArcA

**Figure 3:**
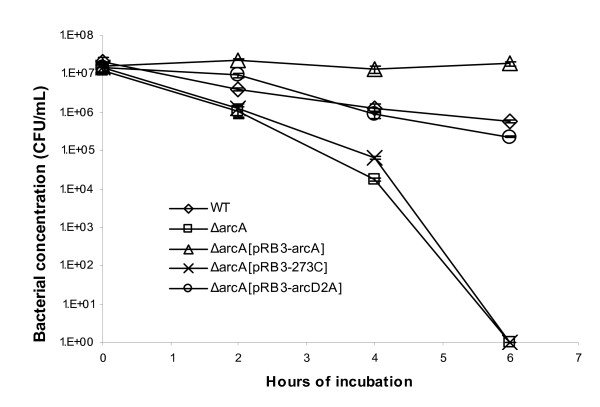
**Plasmid containing phosphorylation-deficient *arcA *complements the *ΔarcA *mutant *E. coli *in resistance to H_2_O_2_**. The wild type *E. coli *(diamond), *ΔarcA *mutant *E. coli *(square), *ΔarcA *mutant *E. coli *transformed with plasmid vector pRB3-273C (cross), *ΔarcA *mutant *E. coli *transformed with plasmid pRB3-arcA (triangle) and *ΔarcA *mutant *E. coli *transformed with plasmid pRB3-arcD2A which contains a phosphorylation-deficient *arcA *allele (circle) were incubated with LB medium containing 1.5 mM H_2_O_2 _at 37°C. The survival of bacteria was determined by plating and plotted against the indicated incubation time period. At least three experiments were performed, and results from a representative experiment performed in triplicates are shown. Error bars indicate standard deviation and sometimes fall within the data label.

Under anaerobic conditions, ArcB is activated by reduced quinones, undergoes auto-phosphorylation, and transfers its phosphorylation to ArcA [25,32,41-43]. It is not known if ArcA is phosphorylated under aerobic conditions or if unphosphorylated ArcA has any function. To test if phosphorylation is necessary for H_2_O_2 _resistance mediated by ArcA, we generated an Asp54 → Ala mutation in ArcA in plasmid pRB3-arcA [38] and used the resulting plasmid pRB3-arcD2A to complement the *ΔarcA *mutant *E. coli*. In H_2_O_2 _resistance assays, plasmid pRB3-arcD2A rescued the *ΔarcA *mutant *E. coli *and the resistance of the mutant to H_2_O_2 _was restored to the wild type level (Figure 3). However, unlike the original plasmid pRB3-arcA, plasmid pRB3-arcD2A did not render the complemented *ΔarcA *mutant *E. coli *more resistant to H_2_O_2 _than the wild type *E. coli *(Figure 3).

### Response of flagellin, OppA and GltI to H_2_O_2 _is altered in the *ΔarcA *mutant *E. coli*

**Figure 4:**
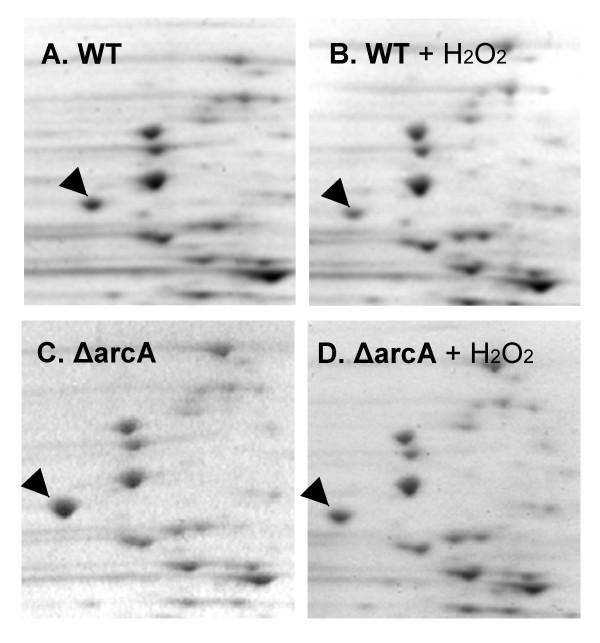
**Two-dimensional gel electrophoresis analysis of whole cell proteins of the wild type and *ΔarcA *mutant *E. coli***. The wild type (WT, A and B) and the *ΔarcA *(ΔarcA, C and D) mutant *E. coli *were exposed to H_2_O_2 _and total proteins from H_2_O_2_-exposed (+H_2_O_2_, B and D) and unexposed bacteria (A and C) were electrophoresed on 2-D gels. Arrows point to the flagellin protein.

To investigate the mechanisms of H_2_O_2 _resistance mediated by ArcA, we performed two-dimensional gel electrophoresis to examine the protein profiles in the *ΔarcA *mutant *E. coli *in the presence or absence of H_2_O_2_, and compared to those of the wild type *E. coli*. While most proteins either were not altered by H_2_O_2 _treatment, or responded similarly to H_2_O_2 _treatment in the wild type and *ΔarcA *mutant *E. coli*, the levels of three proteins were observed to respond to H_2_O_2 _differently, the most abundant of which is shown in Figure 4. By peptide mass fingerprinting using MALDI-TOF, the prominent protein in Figure 4 was identified as flagellin encoded by *fliC*, while the other two less abundant proteins were identified as oligopeptide ABC transporter substrate-binding protein (OppA) and glutamate and aspartate transporter subunit (GltI) (data not shown). The levels of these proteins were quantified in the H_2_O_2_-treated and control untreated samples of the wild type and *ΔarcA *mutant *E. coli *(Table 2).

**Table 2:** Relative levels of differentially regulated proteins in the wild type and *ΔarcA *mutant of *E. coli *K12.

Bacterial strain		Wild type		Δ***arcA***	
**Treatment**		-H_2_O_2_	**+ **H_2_O_2_	-H_2_O_2_	**+ **H_2_O_2_

**Protein**	**FliC**	100	37.9 ± 16.7^†^	188.9 ± 29.8^†^	139.9 ± 57.8^§^
	
	**GltI**	100	2555.5 ± 1343.1^†^	892.0 ± 555.8^†^	440.3 ± 202.2
	
	**OppA**	100	717.5 ± 390.5^†^	205.2 ± 127.3	183.1 ± 67.9

The level of each protein in the untreated wild type *E. coli *is arbitrarily set as 100, and levels of proteins in other samples are expressed as relative to the level in the untreated *E. coli*. Results are the average of three to five independent experiments (biological repeats) with standard deviation. ^† ^Level differs significantly from that of untreated wild type *E. coli*; and ^§ ^level differs significantly from that of wild type *E. coli *treated with H_2_O_2 _(*p *< 0.05, Student's *t*-test).

Flagellin is the only one among the 10 most abundant proteins that responded to H_2_O_2 _treatment. In the wild type, un-treated *E. coli *flagellin was detected at a lower level than in the *ΔarcA *mutant *E. coli*, and H_2_O_2 _treatment further decreased the flagellin level (*p *< 0.05, Student's *t*-test, Table 2 and Figure 4). In the *ΔarcA *mutant *E. coli *H_2_O_2 _treatment also decreased flagellin level, however, the decrease was not statistically significant (Table 2). Therefore, compared to the wild type the *E. coli, ΔarcA *mutant displayed higher flagellin levels both constitutively and following H_2_O_2 _treatment, and its flagellin level did not respond to H_2_O_2 _treatment as that in the wild type *E. coli*.

The response of OppA and GltI expression was different from that of flagellin. In the untreated bacteria levels of both GltI and OppA appeared to be higher in the *ΔarcA *mutant than in the wild type *E. coli *(*p *< 0.05, Student's *t*-test for GltI, Table 2). Following H_2_O_2 _treatment the levels of OppA and GltI in the wild type *E. coli *became higher (*p *< 0.05, Student's *t*-test), while neither protein displayed a statistically significant change in the *ΔarcA *mutant *E. coli *(Table 2). This results in a lower GltI and OppA level in the H_2_O_2 _treated *ΔarcA *mutant than the wild type *E. coli*.

### Flagellin messenger RNA is over-expressed in the *ΔarcA *mutant *E. coli*

**Figure 5:**
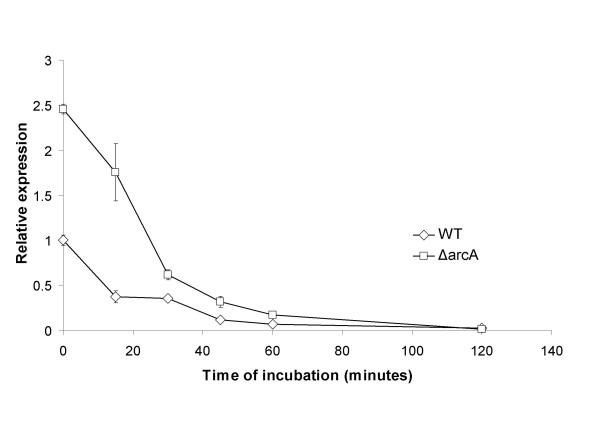
**Expression of *fliC *messenger RNA is regulated in response to H_2_O_2 _exposure**. Expression of *fliC *messenger RNA is regulated in response to H_2_O_2 _exposure. The wild type and the *ΔarcA *mutant *E. coli *was exposed to H_2_O_2_, and the *fliC *messenger RNA in wild type (diamond) and the *ΔarcA *mutant *E. coli *(square) was quantified by Real-time Reverse Transcriptase PCR after various periods of exposure. The level of the *fliC *messenger RNA in the unexposed wild type *E. coli *(at 0 hour) was arbitrarily set as 1, and levels of *fliC *messenger RNA in other samples were expressed as relative expression levels and plotted against the exposure time. At least three experiments were performed, and results from a representative experiment performed in triplicates are shown. Error bars indicate standard deviation.

Since flagellin was one of the most abundant proteins in *E. coli *and the only abundant protein whose level was altered in response to H_2_O_2_, we decided to investigate the influence of flagellin on the survival of the *ΔarcA *mutant *E. coli *in the presence of H_2_O_2_. To determine if the higher protein levels of flagellin in the *ΔarcA *mutant *E. coli *was due to higher levels of mRNA, we examined the expression of the *fliC *transcripts by Real-Time Reverse Transcriptase PCR analysis (RT-PCR). RNA was prepared from the wild type and *ΔarcA *mutant *E. coli *before and after exposure to H_2_O_2_, and subjected to RT-PCR analysis. Similar to protein levels, the *ΔarcA *mutant *E. coli *had higher levels of *fliC *mRNA than the wild type *E. coli *both constitutively and after exposure to H_2_O_2_. In both strains, H_2_O_2_exposure reduced the *fliC *mRNA level progressively (Figure 5). The difference in *fliC *mRNA levels between the wild type and *ΔarcA *mutant *E. coli *decreased with longer exposure periods and no difference could be detected by 120 minutes of exposure (Figure 5). To determine if ArcA directly regulates *fliC *expression, we expressed and purified recombinant ArcA from aerobic cultures of *E. coli *and carried out electrophoretic mobility shift assay of the *fliC *upstream sequence. No specific binding was detected (data not shown).

### Deletion of flagellin increased the survival of the *ΔarcA *mutant *E. coli*

**Figure 6:**
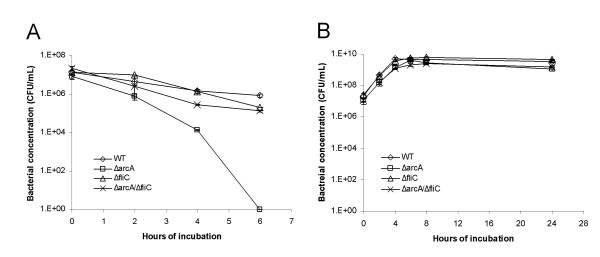
**Deletion of *fliC *increased the resistance of the *ΔarcA *mutant *E. coli *to H_2_O_2_**. Growth and survival of wild type *E. coli *(diamond), *ΔarcA *mutant *E. coli *(square), *ΔfliC *mutant *E. coli *(triangle) and *ΔarcA/ΔfliC *double mutant *E. coli *(cross) in LB medium containing 1.5 mM H_2_O_2 _(a) or LB broth alone (b). The survival of bacteria was determined by plating and plotted against the indicated incubation time period. At least three experiments were performed, and results from a representative experiment performed in triplicates are shown. Error bars indicate standard deviation and sometimes fall within the data label.

Flagellin is one of the most abundant proteins in *E. coli*, and we have shown that its level was higher in the *ΔarcA *mutant *E. coli *both constitutively and upon H_2_O_2 _exposure (Figure 4 and Table 2). We reasoned that expressing an abundant protein such as flagellin at a higher level might be a burden to the *ΔarcA *mutant *E. coli*, especially under stress conditions such as those caused by H_2_O_2_. We hypothesize that a deletion of flagellin encoded by *fliC *may facilitate the survival of the *ΔarcA *mutant *E. coli *exposed to H_2_O_2_. To test this hypothesis, we generated a non-polar *ΔfliC *mutant and an *ΔarcA*/*ΔfliC *double mutant *E. coli*. The non-polar deletion of *fliC *itself had no obvious effect on the survival of *E. coli *in the presence of H_2_O_2 _(Figure 6). However, the *fliC *deletion improved the survival of the *ΔarcA *mutant *E. coli*, and the survival of the *ΔarcA*/*ΔfliC *double mutant *E. coli *was close to that of the wild type *E. coli *(Figure 6). This indicates that elimination of flagellin in the *ΔarcA *mutant *E. coli *enhanced its survival under H_2_O_2 _stress.

In addition to flagellin, we have also attempted to delete other abundant proteins to determine if such deletions would improve the survival of the *arcA *mutant *E. coli*. Our efforts were not successful, however, because most abundant proteins such as elongation factors, 30 s ribosomal proteins, and chaperone proteins are either essential or important for *E. coli*, and such deletions would be detrimental to *E. coli*. We successfully deleted D-ribose periplasmic binding protein (RbsB) encoded by *rbsB*, a protein which is as abundant as or more abundant than flagellin. The *ΔrbsB *mutant itself was found to be susceptible to H_2_O_2_, therefore could not be used to test the effect of RbsB on the H_2_O_2 _resistance of the *arcA *mutant *E. coli *(data not shown).

### Amino acid supplementation improved the survival of the *ΔarcA *mutant *E. coli *under H_2_O_2 _stress

**Figure 7:**
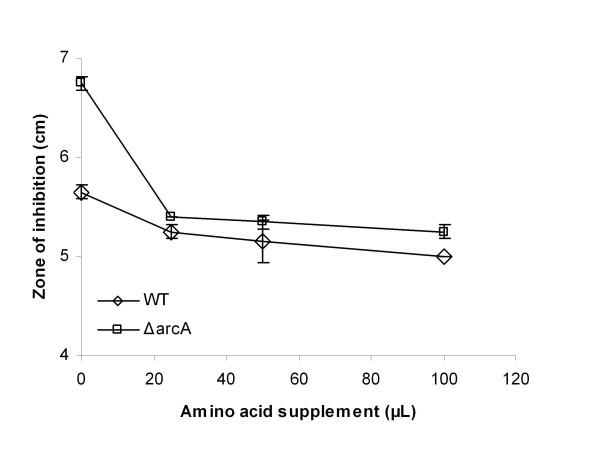
**Amino acid complementation increased the resistance of *E. coli *to H_2_O_2 _and reduced the difference in H_2_O_2 _resistance between the wild type and *ΔarcA *mutant *E. coli***. Resistance of wild type (diamond) and the *ΔarcA *mutant *E. coli *(square) to H_2_O_2 _was assayed by the ability to grow in the presence of H_2_O_2 _and more resistant bacteria show a smaller diameter of inhibition. Various volumes of 20 mM amino acid solution was spread onto each M9 minimal medium plate containing approximately 1 × 10^6 ^c.f.u. wild type or *ΔarcA *mutant *E. coli *and a paper disc of 1/4" with 10 μl of 30% H_2_O_2 _was added to the center of each plate. Zone of inhibition was measured after overnight incubation and plotted against the volume of amino acid supplementation. At least three experiments were performed, and results from a representative experiment performed in triplicates are shown. Error bars indicate standard deviation and sometimes fall within the data label..

We described above that a deletion of flagellin in *E. coli *improved the survival of the *ΔarcA *mutant *E. coli *in the presence of H_2_O_2_. Our analysis of the proteome of the wild type and *ΔarcA *mutant *E. coli *indicated that levels of glutamine/aspartate periplasmic binding protein (GltI) and oligopeptide binding protein precursor (OppA) increased in the *ΔarcA *mutant as compared to the wild type *E. coli *(Table 2). In addition, the *ΔarcA *mutant *E. coli *failed to increase GltI and OppA protein levels in response to H_2_O_2 _as the wild type *E. coli*. This suggests that *E. coli *may have an increased need for amino acids under H_2_O_2 _stress and the *ΔarcA *mutant *E. coli *may benefit from amino acid supplementation. To test this hypothesis, we determined the effect of amino acid supplementation on the survival of the *ΔarcA *mutant *E. coli *in the presence of H_2_O_2_. To facilitate a direct comparison between the resistance of the wild type and *ΔarcA *mutant *E. coli *to H_2_O_2 _with or without amino acid supplementation, we carried out a disc diffusion assay, and bacterial resistance to H_2_O_2 _was measured by the diameter of the zone of inhibition (ZOI). Without amino acid supplementation the ZOI of the *ΔarcA *mutant *E. coli *was significantly larger than that of the wild type *E. coli *(Figure 7). With amino acid supplementation, sizes of the ZOI reduced for both the wild type and the *ΔarcA *mutant *E. coli*, and the difference in the sizes of the ZOI between wild type and *ΔarcA *mutant *E. coli *diminished with amino acid supplementation (Figure 7). We tested single amino acids and combinations of various amino acids, and none of the combinations tested was able to complement the susceptibility of the *ΔarcA *mutant *E. coli *as the total amino acids (data not shown).

### Antibiotic that inhibits protein synthesis increased susceptibility of *E. coli *to H_2_O_2_

**Figure 8:**
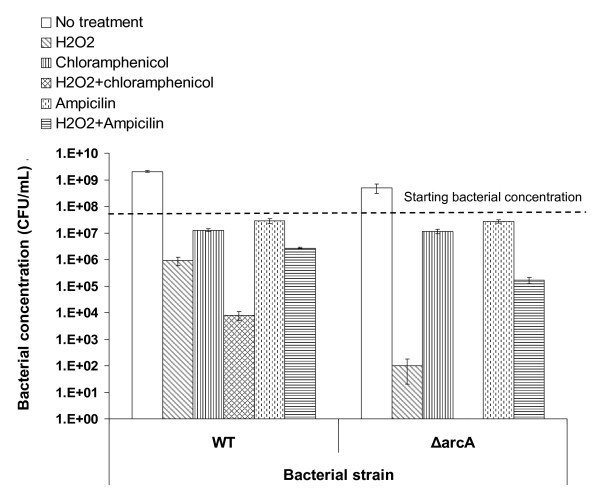
**Chloramphenicol enhanced the bactericidal activity of H_2_O_2_**. The wild type *E. coli *(WT) and the *ΔarcA *mutant *E. coli *(ΔarcA) were incubated in M9 minimal medium containing 1.5 mM H_2_O_2 _for 6 hours at 37°C. The survival of bacteria was determined by plating. Bacterial concentration following each treatment (open bars, no treatment; diagonally-hatched bars, H_2_O_2_; vertically-hatched bars, 25 μg ml^-1 ^of chloramphenicol; cross-hatched bars, H_2_O_2 _and 25 μg ml^-1 ^of chloramphenicol; dotted line-hatched bars, 50 μg ml^-1 ^ampicillin and horizontally-hatched bars, H_2_O_2 _and 50 μg ml^-1 ^ampicillin) was plotted on the graph. The horizontal dashed line indicates the starting concentration of bacteria.

To test if protein synthesis is important for bacterial survival and if protein synthesis inhibition is detrimental to bacteria under reactive oxygen stress, we assayed the resistance of *E. coli *to H_2_O_2 _in the presence of chloramphenicol, an antibiotic that inhibits peptide bond formation and hence protein synthesis. Without H_2_O_2 _or antibiotic, wild type *E. coli *grew approximately 2log_10 _during 6 hours of incubation (Figure 8, left half, open bar). Hydrogen peroxide was bactericidal and the bacterial concentration decreased for over 1_log10 _(Figure 8, left half, diagonally-hatched bar). Supplementation of chloramphenicol alone prohibited bacterial proliferation and the bacterial concentration decreased slightly (Figure 8, left half, vertically-hatched bar). Incubation in the presence of both H_2_O_2 _and chloramphenicol was more detrimental to *E. coli *than either H_2_O_2 _or chloramphenicol alone, and the bacterial concentration decreased by nearly 4log_10 _(Figure 8, left half, cross-hatched bar). This indicates that chloramphenicol enhanced the bactericidal activity of H_2_O_2_. To determine if this enhanced bactericidal activity is due to the bacteriostatic activity of chloramphenicol, we tested the effect of ampicillin, an antibiotic that inhibits the bacterial cell wall synthesis, in the same assay. When added alone, ampicillin had similar effect on bacterial growth as chloramphenicol did (Figure 8, left half, dotted line-filled bar). However, in contrast to chloramphenicol that enhanced the bactericidal effect of H_2_O_2 _(Figure 8, left half, cross-hatched bar), the addition of ampicillin reduced the bactericidal activity of H_2_O_2 _for unknown reasons (Figure 8, left half, compare horizontally hatched bar to diagonally-hatched bar). This indicates that the synergistic effect of chloramphenicol on the bactericidal activity of H_2_O_2 _is not due to its bacteriostatic effect and suggests that protein synthesis is important for *E. coli *to resist the killing by H_2_O_2_.

Similar assays were carried out with the *ΔarcA *mutant *E. coli *and the results were consistent with those of the wild type *E. coli*. While incubation with H_2_O_2 _alone reduced the concentration of the *ΔarcA *mutant *E. coli *by over 5log_10 _after 6 hours of incubation (Figure 8, right half, diagonally-hatched bar), the addition of chloramphenicol to the assay eliminated all *E. coli *(Figure 8). The synergistic effect of the bactericidal activity of H_2_O_2 _and chloramphenicol on the *ΔarcA *mutant *E. coli *is not because it is more susceptible to chloramphenicol (Figure 8, vertically-hatched bars). Similarly to that observed with wild type *E. coli*, ampicillin reduced the bactericidal activity of H_2_O_2_, and the *ΔarcA *mutant *E. coli *survived better in the presence of both ampicillin and H_2_O_2 _than H_2_O_2 _alone (1.7 × 10^5 ^CFU/ml vs. 1.0 × 10^2 ^CFU/ml) (Figure 8).

## Discussion

Although the ArcAB system has been extensively investigated for its role as the global control system of *E. coli *in anaerobic growth, its role, if any, in aerobic growth is much less understood. We have previously reported that ArcA is necessary for the pathogenic bacterium *Salmonella enterica *to resist reactive oxygen and nitrogen species under aerobic conditions [38]. In this report, we used *E. coli *as our model to further explore the role of both ArcA and ArcB in ROS resistance, and to investigate the mechanism of ROS resistance mediated by the ArcAB two-component system. Here we demonstrate that deletion mutants of ArcA and ArcB were more susceptible to H_2_O_2_, suggesting that both ArcA and ArcB were necessary for *E. coli *to resist the stress caused by H_2_O_2 _(Figure 1), and that their functions were not limited to anaerobic growth of bacteria. Interestingly, we have not detected any growth defects of *ΔarcA *or *ΔarcB *mutant *E. coli *under anaerobic conditions (data not shown) and to our knowledge no such defect has been reported in the literature. In addition, an *ΔarcA *mutant of *Salmonella enterica *grew normally in anaerobic medium [38]. This further indicates that ArcAB has wider roles in the physiology and metabolism of enteric bacteria besides its well-characterized regulation of anaerobic growth of bacteria.

The signaling pathway of the ArcAB system under anaerobic conditions has been extensively characterized [25-28,30-34,42,44]. The membrane-bound sensor-kinase ArcB is activated by reduced quinones under anaerobic conditions, and subsequently activates its cognate transcriptional regulator ArcA by phosphorylating ArcA at Asp54 [30,42,25]. Matsushika and Mizuno previously reported that ArcB can also phosphorylate ArcA directly through His292 under aerobic conditions [45], however, its physiological relevance to *E. coli *has not been reported. Our results on the role of ArcAB in ROS resistance suggest that ArcAB can be activated by novel signals other than reduced quinones and anaerobic conditions, and the activation is independent of phosphorylation at Asp54 of ArcA as demonstrated under anaerobic conditions [41,42,46], since phosphorylation-defective ArcA expressed from a plasmid fully complemented an *ΔarcA *mutant *E. coli *for its susceptibility to H_2_O_2 _(Figure 3). We would like to point out that our analysis was conducted using a phosphorylation-mutant ArcA (Asp54 → Ala) expressed from a plasmid. It is yet to be determined if a mutant carrying a corresponding mutation of *arcA *in the chromosome is susceptible to H_2_O_2_. (Our attempts to generate a mutant *arcA *encoding an Asp54 → Ala mutation in the chromosome were unsuccessful due to technical difficulties. Similar to what we observed for *arcB*, plasmids carrying *arcA *were prone to mutations during cloning.) We have also noticed that the wild type ArcA expressed from a plasmid confers a stronger H_2_O_2 _resistance phenotype than the phosphorylation-defective ArcA. The *ΔarcA *mutant *E. coli *complemented *in trans *with a wild type *arcA *allele demonstrated higher H_2_O_2 _resistance than the wild type *E. coli *(Figure 1 and 3), while the same mutant *E. coli *complemented with a phosphorylation-defective *arcA *allele has the same H_2_O_2 _resistance as the wild type *E. coli *(Figure 3).

In addition to novel signals and signaling pathways that may mediate the function of the ArcAB system in the ROS resistance, the ArcAB system may also regulate a distinct set of genes under aerobic conditions. Under anaerobic conditions ArcA mostly negatively regulates genes involved in the TCA cycle and electron transport [26-28]. Under aerobic conditions, a microarray study by Oshima *et al*. demonstrated that expression of a large number of genes in the *ΔarcA *or *ΔarcB *mutant *E. coli *was altered [23]. Our results suggest that levels of at least three proteins (flagellin, GltI and OppA) were altered in the *ΔarcA *mutant *E. coli *both constitutively and in response to H_2_O_2 _treatment (Figure 4 and Table 2). Our further analysis on the messenger RNA level of *fliC *indicates that the RNA levels are higher in the *ΔarcA *mutant *E. coli *and corresponded to the protein levels, suggesting that the regulation is likely on the transcriptional or post-transcriptional level (Figure 5). Oshima *et al*. did not detect a significant alteration in the expression of *fliC *in their microarray analysis, although flagellar synthesis was identified as a system that was affected in the *ΔarcA *mutant but not the *ΔarcB *mutant *E. coli *[23]. The discrepancy is possibly due to the differences in experimental conditions (shaking bacterial cultures at 120 rpm vs. 225 rpm) and detection methods (microarray vs. Real-Time Reverse Transcriptase PCR and 2-D gel electrophoresis). Since we detected an elevation of both mRNA and protein levels of flagellin in the *ΔarcA *mutant *E. coli *(Figures 4 and 5), we believe that our observation is valid. The regulation of ArcA on flagellin is likely to be indirect, as we did not detect specific binding of recombinant ArcA protein to the upstream sequence of *fliC *(data not shown).

Given that the ArcAB system regulates a large number of genes in *E. coli*, its role in the ROS resistance is likely to be complex. We have demonstrated that mutation of ArcA or ArcB did not alter the H_2_O_2 _scavenging ability of *E. coli *(Figure 2), however, the precise molecular mechanism on how ArcA regulates ROS resistance in *E. coli *is yet to be elucidated. ArcA was reported to be necessary for the ROS resistance of *Haemophilus influenzae *due to its regulation of Dps, a ferritin-like small protein that was previously reported to be involved in ROS resistance of *Salmonella *[39,47]. The mechanism of the ROS resistance mediated by ArcA is likely to be different in *E. coli*, since *dps *is expressed close to the wild type level in the *ΔarcA *or *ΔarcB *mutant (84% and 99% respectively), and our preliminary microarray analysis with *Salmonella ΔarcA *mutant indicated that *dps *responded normally to H_2_O_2 _in the *ΔarcA *mutant (unpublished results). One possible clue on the mechanism of how ArcAB contributes to the ROS resistance of *E. coli *came from our proteomic analysis that showed altered expression of flagellin, GltI and OppA between the wild type and *ΔarcA *mutant *E. coli *(Table 2). The constitutive GltI and OppA levels are higher in the *ΔarcA *mutant than in the wild type *E. coli*, suggesting that the mutant may have a higher need for amino acid transport. In contrast to the GltI and OppA levels in the wild type *E. coli *that increased 6- and 24-fold respectively in response to H_2_O_2 _exposure (possibly due to a higher need for amino acid transport under ROS stress), the level of neither protein in the *ΔarcA *mutant increased under the same condition (Table 2). A higher level of flagellin in the *ΔarcA *mutant likely put further constraint on the protein synthesis, and as a result the *ΔarcA *mutant *E. coli *might have become less fit under H_2_O_2 _stress. Our genetic study demonstrating that deletion of *fliC *"rescued" the survival defect of the *ΔarcA *mutant *E. coli *under H_2_O_2 _stress (Figure 6) supports the hypothesis.

ROS stress conditions induce growth arrest in *E. coli*. Chang *et al*. has reported that in growth arrest induced by either glucose-lactose diauxie, entry into stationary phase, or H_2_O_2 _treatment, genes involved in amino acid biosynthesis pathways are down-regulated except those of histidine and arginine biosynthesis [24]. Recently, Jang and Imlay have shown that H_2_O_2 _damages enzymes with iron-sulfur and impairs bacterial metabolism, especially the biosynthesis of leucine [48]. This down regulation of amino acid synthesis may cause a strain on the protein synthesis of bacteria. Our results indicate that protein synthesis is important for *E. coli *to survive H_2_O_2 _treatment. Chloramphenicol, an antibiotic inhibiting protein synthesis, reduced the survival of both the wild type and *ΔarcA *mutant *E. coli *after H_2_O_2 _treatment, while ampicillin did not (Figure 8). Consistently, amino acid supplementation enhanced the survival of *E. coli *after H_2_O_2 _treatment (Figure 7). This is in agreement with the report by Calioz and Touati that amino acid supplementation facilitates the survival of superoxide dismutase-deficient *E. coli *under aerobic conditions [49].

Although our results and results from other investigators suggest that protein synthesis and amino acid availability are important for *E. coli *to survive ROS stress and the global regulatory system ArcAB plays a role this aspect of ROS stress resistance, protein synthesis and amino acid availability may be only one aspect of the pleiotropic effect of ArcAB system on *E. coli*, since chloramphenicol-treated *ΔarcA *mutant was still more susceptible than the similarly treated wild type *E. coli*. Further studies are necessary to elucidate more molecular mechanisms that control the ROS resistance mediated by the ArcAB global regulatory system.

## Conclusion

The global regulatory system ArcAB of *E. coli *regulate many important functions of bacteria including anaerobic growth, motility, and cell division. Here we demonstrate that ArcAB regulates ROS resistance under aerobic condition, and the signalling pathway of this regulation is distinct from that under anaerobic conditions. The ArcAB system may regulate protein and amino acid synthesis and transport that influence the fitness of *E. coli *under ROS stress.

## Methods

### Reagents

Growth media for bacteria were purchased from Becton Dickinson and Company (Franklin Lakes, NJ). Anaerobic peptone-yeast medium was obtained from Anaerobe Systems (Morgan Hills, CA). Chemicals and antibiotics were purchased from Sigma-Aldrich Chemical Co. (St. Louis, MO) unless otherwise indicated. Restriction and modifying enzymes for manipulating DNA were purchased from the New England Biolabs (Beverly, MA). Custom oligonucleotides were purchased from Sigma Genosys (The Woodlands, TX).

### Bacterial strains and plasmids

*E. coli *strain K12 isolate MG1655 (gift from Dr. Sydney Kustu, University of California) was used as the parental strain in all analyses described in this report. Mutagenesis was carried out using the one-step mutagenesis method by Datsenko and Wanner [50]. Mutant bacterial strains and sequences of oligonucleotides used for mutagenesis are listed in Table 1. In the *ΔarcA *mutant, the wild type *arcA *allele was replaced by a kanamycin-resistance cassette (Kan^r^). In the *ΔarcB *mutant, the wild type *arcB *allele was replaced by a chloramphenicol-resistance cassette (Cm^r^). Each mutation was transduced into fresh *E. coli *by general transduction with phage P1 before further analysis. In the *ΔfliC *mutant, the wild type *fliC *allele was replaced by Cm^r^, which was subsequently removed to generate a non-polar mutant [50]. The *ΔarcA*/*ΔfliC *mutant was prepared by transducing *arcA*::*kan *from the *ΔarcA *mutant into the *ΔfliC *non-polar mutant *E. coli*. A revertant of *ΔarcB *mutant *E. coli *was generated through a two-step process. First, a mutant, *arcB*(Kan^r^), was generated in which Kan^r ^was inserted downstream to the *arcB *coding sequence without affecting the *arcB *open reading frame. Subsequently, phage P1 was prepared from *arcB*(Kan^r^) and used to transduce the *ΔarcB *mutant *E. coli*. Kanamycin-resistant and chloramphenicol-sensitive colonies were selected, in which the deletion mutant *arcB *allele in the *ΔarcB *mutant *E. coli *was replaced by a wild type allele from *arcB*(Kan^r^). The genome structure surrounding the *arcB *allele was determined to verify that wild type *arcB *allele was restored. The resultant bacterial strain was referred to as *ΔarcB*-rev.

Plasmid pRB3-arcA used to complement the *ΔarcA *mutant *E. coli *was described previously [38]. Plasmid pRB3-arcD2A was constructed using megaprimer method as described [51]. Briefly, a 260-bp section of the *arcA *gene that included the Asp54 was amplified using mutagenesis primer 5'-CAACCTGGTGATCATG**GCG**ATCAATCTGCC-3' and an *arcA *primer 5'-CAACGCTACGACGCTCTTC-3'. Sequence in bold in the mutagenesis primer introduced an aspartate to alanine mutation (Asp → Ala) at amino acid 54 in ArcA. The PCR product was used as a megaprimer to amplify plasmid pRB3-arcA together with a vector primer 5'-GTTTTCCCAGTCACGAC-3'. The PCR product was subsequently digested with KpnI and cloned into KpnI-digested plasmid pRB3-arcA to replace the wild type *arcA *gene with the corresponding sequence that introduced an Asp54 → Ala mutation. The resulting plasmid pRB3-arcD2A contained the same sequence as the original plasmid pRB3-arcA except that GAT which codes for Asp54 of ArcA was mutated to GCG which codes for Ala.

### Survival assays of bacteria after exposure to oxidative and other stresses

Survival of *E. coli *after H_2_O_2 _and other stress conditions was assayed as described previously [38,52]. *E. coli *was cultured in 2 ml of Luria Bertani (LB) broth at 37°C overnight with shaking at 225 rpm. Antibiotics were added as appropriate. Twenty microliters of overnight cultures were added to 2 ml of LB containing one of the following chemicals: hydrogen peroxide, sodium chloride, or sodium dodecyl sulfate (SDS). Cultures in all assays were grown aerobically by shaking at 225 rpm. After exposure to H_2_O_2 _or other stresses, aliquots of cultures were diluted and plated in triplicates. Bacterial colonies were enumerated as colony-forming units (CFU) after overnight incubation to determine the bacterial concentration. Disc diffusion assay was carried out as described previously [52]. Briefly, approximately 1 × 10^6 ^cfu bacteria were plated onto M9 minimal agar plates and paper discs of 1/4" diameter loaded with 10 μl of 30% H_2_O_2 _were placed in the center of plates onto the bacterial lawn. Plates were incubated overnight at 37°C, and the diameter of the inhibitory zone on each plate was measured.

### Scavenging of H_2_O_2 _by *E. coli*

Wild type, the *ΔarcA *and the *ΔarcB *mutant *E. coli *were cultured overnight in LB broth at 37°C with shaking at 225 rpm. Twenty microliters of overnight bacterial culture was diluted in 1 mL of fresh LB broth containing 2 mM of H_2_O_2 _that had been pre-warmed to 37°C. An aliquot of 100 μL was taken as the 0 minute sample, and rest of the cultures were incubated at 37°C with shaking. Subsequently, aliquots were taken at 10' intervals. Aliquots of bacterial cultures were used for plating to determine the bacterial concentration, and the rest of the samples were used to determine the concentration of H_2_O_2_. A control sample of LB supplemented with H_2_O_2 _that contained no bacteria was included in all assays for spontaneous degradation of H_2_O_2_.

The concentration of H_2_O_2 _in bacterial cultures was determined as described [53]. Briefly, bacterial cultures were spun down to remove bacteria and 40 μL of supernatant was diluted in 260 μL of 50 mM potassium phosphate (pH7.0). Diluted supernatant was mixed with 600 μL of a reaction mixture containing 500 nM H_2_O_2_, 2.5 mM phenol, 0.5 mM 4-aminoantipyrine, 40 μg horseradish peroxidase, and 1 mM potassium phosphate (pH 7.0) [53]. The reactions were incubated at room temperature for approximately 10' till color stabilized, and OD_505 nm _was measured for each sample. The concentration of H_2_O_2 _was determined by a standard curve generated with known concentrations of H_2_O_2 _in LB broth. The H_2_O_2 _scavenging was determined as (initial H_2_O_2 _concentration – residual H_2_O_2 _concentration) (in mM)/bacterial concentration (in 10^7 ^cfu/mL).

### Real-Time Reverse Transcriptase Polymerase Chain Reaction (RT-PCR) analysis of gene expression

To analyze the expression of *fliC *messenger RNA, we cultured the wild type and *ΔarcA *mutant *E. coli *in LB broth to log phase and divided each culture into two aliquots. One of the aliquots was exposed to 5 mM H_2_O_2 _and samples were taken after different exposure periods. The other aliquot was used as an unexposed control. Total RNA was purified from *E. coli *using TRIzol reagent (Invitrogen, Carlsbad, CA) followed by digestion with DNase I (Qiagen, Valencia, CA) and purification by RNeasy kit (Qiagen, Valencia, CA). Subsequently, 1.5 μg RNA were reverse-transcribed using M-MLV reverse transcriptase (Promega, Madison, WI), and cDNA samples were used for Real-Time Reverse Transcriptase PCR analysis (RT-PCR). RT-PCR was performed using the iQ SYBR Green PCR supermix (Bio-Rad, Hercules, CA) in an iCycler (Bio-Rad, Hercules, CA). Primers 5'-GGCGGAACTAACCCAGCTTCA-3' and 5'-TGCTCCAGTCGCCATTGTCA-3' were used for the RT-PCR analysis of *fliC *expression. The 16S ribosomal RNA level was determined with primers 5'-GGGACCTTCGGGCCTCTTG-3' and 5'-ACCGTGTCTCAGTTCCAGTGTGG-3', and was used to normalize expression levels of *fliC *from different samples.

Q-Gene program and Relative Expression Software Tool (REST) were used for data analysis of threshold cycle numbers from the iCycler [54,55]. Mean values of normalized expression and standard error measurements were determined as described [54]. Comparisons of mean normalized expression were used to calculate expression ratios. REST was used to obtain statistical significance (*p*-value) as described [55].

### Bacterial extracts and two-dimensional (2-D) gel electrophoresis

*E. coli *was cultured in LB broth overnight at 37°C with shaking. Overnight bacterial culture was diluted 1:100 in fresh LB and cultured for 4 hours at 37°C with shaking, and then split into two aliquots. Hydrogen peroxide was added to 5 mM to one of the aliquots, and both aliquots were further incubated for 2 hours at 37°C with shaking. Bacterial cultures were chilled on ice immediately and spun down. Bacterial pellets were then resuspended in 8 M urea and 4% CHAPS in 10 mM Tris 8.0 and sonicated. The insoluble fraction was removed by centrifugation, and soluble lysate was used for 2-D gel electrophoresis.

Two-dimensional gel electrophoresis of *E. coli *proteins was performed with the Zoom IPG Runner system following the manufacturer's instructions (Invitrogen, Carlsbad, CA). One hundred fifty micrograms of cellular proteins were diluted in rehydration buffer (8 M urea, 4% CHAPS and 0.5% pH 3–10 ampholytes) and loaded onto each pH 3–10 ZOOM strip (Invitrogen, Carlsbad, CA). The first dimension electrophoresis was carried out at 200 V for 20', 450 V for 15', 750 V for 15' and 2000 V for 60'. After isoelectric focusing, ZOOM strips were reduced and alkylated with 125 mM iodoacetamide and electrophoresed on NuPAGE Novex 4–12% Bis-Tris ZOOM gels (Invitrogen, Carlsbad, CA) at 100 V for 90'. Proteins were visualized by staining with ProteomIQ reagents (Proteome Systems, Woburn, MA), and then scanned with a HP Scanjet 5530 scanner (Hewlett-Packard, Palo Alto, CA). Individual proteins were quantified using ImageQuant (Amersham Biosciences, Piscataway, NJ) and normalized against the total protein content of the gel.

### Mass spectrometry analysis of protein spots

Protein spots of interest were excised from gels and washed with 50% acetonitrile in 50 mM ammonium bicarbonate twice for 15' each. The gel spots were then dehydrated in acetonitrile for 30' and dried in a speed vac for 10'. Thirty microliters of 50 mM ammonium bicarbonate containing 0.3 μg of trypsin (Sigma-Aldrich, St Louis, MO) were added to each sample, and samples were incubated at 37°C for 16 hours. Digested peptides were extracted from gel spots by two washes of 50% acetonitrile/0.1% trifluoroacetic acid, and purified with Ziptips (Millipore, Billerica, MA). Purified peptides were eluted from Ziptips with 50% acetonitrile/0.05% trifluoroacetic acid with 10 mg/ml alpha-cyano-4-hydroxycinnamic acid, and spotted on a sample plate to obtain mass spectra using an Axima CFR Plus MALDI-ToF mass spectrometer (Shimadzu Biotech, Columbia, MD). Each spectrum was calibrated externally using the ProteoMass peptide MALDI-MS calibration kit (Sigma-Aldrich, St Louis, MO).

Peptide fingerprints obtained for each sample were used to search the databases at NCBI and SWISS-PROT using MASCOT search engine http://www.Matrixscience.com. Search parameters used were variable carbamidomethyl and propionamide modifications of cysteines and oxidation of methionines. A peptide tolerance window of 0.5 daltons was used for all searches. Once an identification was made with a statistically significant score, data were accepted when the peptide coverage of the protein was at least 20%, and the molecular weight and isoelectric point of the protein matched those observed on the 2D gel electrophoresis.

## Authors' contributions

CL participated in the study design, carried out the microbiological studies and helped to draft the manuscript. AC carried out the microbiological studies. SL conceived of the study, participated in the study design, carried out the microbiological studies, performed the statistical analysis and drafted the manuscript. All authors read and approved the final manuscript..
